# Assessment of Genetic Correlation between Bacterial Cold Water Disease Resistance and Spleen Index in a Domesticated Population of Rainbow Trout: Identification of QTL on Chromosome Omy19

**DOI:** 10.1371/journal.pone.0075749

**Published:** 2013-10-09

**Authors:** Gregory D. Wiens, Roger L. Vallejo, Timothy D. Leeds, Yniv Palti, Sima Hadidi, Sixin Liu, Jason P. Evenhuis, Timothy J. Welch, Caird E. Rexroad

**Affiliations:** National Center for Cool and Cold Water Aquaculture, Agricultural Research Service, United States Department of Agriculture, Kearneysville, West Virginia, United States of America; INRA, France

## Abstract

Selective breeding of animals for increased disease resistance is an effective strategy to reduce mortality in aquaculture. However, implementation of selective breeding programs is limited by an incomplete understanding of host resistance traits. We previously reported results of a rainbow trout selection program that demonstrated increased survival following challenge with *Flavobacterium psychrophilum*, the causative agent of bacterial cold water disease (BCWD). Mechanistic study of disease resistance identified a positive phenotypic correlation between post-challenge survival and spleen somatic-index (SI). Herein, we investigated the hypothesis of a genetic correlation between the two traits influenced by colocalizing QTL. We evaluated the inheritance and calculated the genetic correlation in five year-classes of odd- and even-year breeding lines. A total of 322 pedigreed families (n = 25,369 fish) were measured for disease resistance, and 251 families (n = 5,645 fish) were evaluated for SI. Spleen index was moderately heritable in both even-year (*h^2^* = 0.56±0.18) and odd-year (*h^2^* = 0.60±0.15) lines. A significant genetic correlation between SI and BCWD resistance was observed in the even-year line (*r_g_* = 0.45±0.20, *P* = 0.03) but not in the odd-year line (*r_g_* = 0.16±0.12, *P* = 0.19). Complex segregation analyses of the even-year line provided evidence of genes with major effect on SI, and a genome scan of a single family, 2008132, detected three significant QTL on chromosomes Omy19, 16 and 5, in addition to ten suggestive QTL. A separate chromosome scan for disease resistance in family 2008132 identified a significant BCWD QTL on Omy19 that was associated with time to death and percent survival. In family 2008132, Omy19 microsatellite alleles that associated with higher disease resistance also associated with increased spleen size raising the hypothesis that closely linked QTL contribute to the correlation between these traits. To our knowledge, this is the first estimation of spleen size heritability and evidence for genetic linkage with specific disease resistance in a teleost fish.

## Introduction

Bacterial cold-water disease (**BCWD**) is an important disease impacting salmonid aquaculture. The etiological agent of BCWD is a gram-negative bacterium, *Flavobacterium psychrophilum,* which also causes rainbow trout fry syndrome (RTFS) in small fish [1], [2], [3]. Economic losses from *F. psychrophilum* are due to direct mortality and also to deformities in fish that survive infection [2], [4]. The pathogen is widely distributed and disease prevention through biosecurity is not currently feasible. Salmonids can be infected at early life stages prior to typical vaccination [2], [5], [6], [7], and at present, no licensed commercial vaccine is available for BCWD or RTFS in the U.S. Host genetic variation for survival following *F. psychrophilum* challenge has been demonstrated in rainbow trout [8], [9], [10] indicating a favorable potential for family-based selective breeding to reduce BCWD.

We have previously reported the establishment of a selective breeding program to increase rainbow trout genetic resistance against BCWD [10]. BCWD post-challenge survival is a moderately heritable trait that was not adversely correlated with growth performance [10]. Furthermore, we found that most families maintained their relative resistant or susceptible phenotype as average body weight increased over 300-fold [11]. These results demonstrated that the resistance mechanism(s) were not expressed in a transitory manner during development. In these studies, unexposed resistant families displayed larger-than-average spleen size normalized to body weight (spleen somatic-index, **SI**), while susceptible families had smaller-than-average SI. This observation was of interest as mammalian spleen size has been correlated with clearance capacity, differences in immune cell subsets, and resistance to infection by encapsulated bacteria (11). In our studies, following challenge, the SI of resistant families was larger than the susceptible families on days 3 and 5 post-infection. Furthermore, resistant fish had a 14-fold lower bacterial load in the spleen on day 5 post-challenge, indicating a greater capacity to limit bacterial growth or trafficking following challenge. The association between resistance and SI was further confirmed in families from a separate population where families were first phenotyped for SI, and this was later found to predict *F. psychrophilum* post-challenge survival [11]. Based on the results from these separate populations, we postulated a genetic link between rainbow trout survival following *F. psychrophilum* challenge and SI.

In order to understand the genetic control of BCWD resistance, we tested the hypothesis herein that colocalizing (closely-linked) loci influence both spleen size and post-challenge survival by quantifying the genetic correlation between both traits over five year classes using a large number of pedigreed families. In addition, siblings from families with extreme phenotypes were mated and these fish were used to determine the stability of family-mean SI during fish growth. From these data a model of inheritance is proposed based on parent to offspring transmission and complex segregation analyses using phenotypic data. Using molecular markers in a genome scan, we report the identification of multiple QTL influencing SI and provide evidence that one of these loci, located on Omy19, is closely linked to BCWD resistance. The number, genomic positions, and effects of significant QTL and their contribution to phenotypic variance of SI and BCWD resistance are described.

## Materials and Methods

### Ethics Statement

All fish were maintained at the National Center for Cool and Cold Water Aquaculture (NCCCWA) following NCCCWA Standard Operating Procedures for the Care and Use of Research Animals (Rainbow trout). The NCCCWA IACUC committee approved this study (Protocols #029, 033, 047 and 053). Steps were taken to ameliorate suffering in all work involving experimental fish. Each year, rainbow trout were spawned in late December through February and the embryos were developmentally-synchronized using water temperature manipulation in order to synchronize hatch dates within an approximate 2-week period in late February or early March as previously described [9]. Samples from random fish representing the five year classes were screened for reportable pathogens in addition to *F. psychrophilum* and found to be negative as previously described [12]. Post-hatch, families were maintained in separate tanks receiving 12–14°C flow-through well and spring water and fed a standard commercial diet (Ziegler Bros, Inc., Gardners, PA). The pellet size and percent body weight were adjusted according to NCCCWA standard feeding protocol for rainbow trout and fish were maintained at biomass densities <50 kg/m^3^. After growth to 15 g, feed was delivered to each tank using Arvotec (Huutokoski, Finland) automatic feeders. Tricaine methanesulfonate, MS 222 (Sigma) was used to anesthetize the fish at 100 mg L^−1^ for routine handling and weighing; while for lethal sampling, fish were sacrificed using a 200 mg L^−1^ concentration for at least 5 minutes.

### Phenotyping Methods

In general, fish were disease challenged at the smallest feasible size as BCWD farm mortality is typically highest when fish are between 0.2 to 4 g body weights. Families were maintained in separate tanks and evaluated for survival in seven challenge experiments (Table S1 in File S1, and Data S1 in File S2). A total of 322 defined-pedigree families (n = 25,369 fish) were measured for BCWD survival at sizes averaging from 2.4 g to 39.6 g and ages from 82 to 190 days post-hatch (Table S1 in File S1). *Flavobacterium psychrophilum,* strain CSF 259-93, was used for all challenges and was initially provided by Dr. S. LaPatra (Clear Springs Foods, Inc.). This strain is commonly utilized in challenge studies [13], [14], [15] and the clone used in this study was selected and stored as previously described [12]. Briefly, thawed bacteria were grown on TYES plates at 15°C for 5 days, and after suspension of bacteria in PBS, each fish was injected through the base of the pelvic fin and into the peritoneal cavity with *F. psychrophilum* cells or PBS alone. Challenge dose and injection volume were determined based on the fish population average body weight. The number of viable bacteria injected per fish was determined by plate count after fish challenge and ranged from 0.2×10^6^ to 3.8×10^6^ CFU per g body weight (Table S1 in File S1). Fish were examined for typical clinical signs of disease including injection site hemorrhage, tissue necrosis and associated lesion(s), and a limited number of moribund or dead fish were examined using either gram stain or plate culture to confirm the presence of the challenge pathogen. Mortality in PBS-injected fish was negligible (<3%) demonstrating that mortality was specific to the challenge pathogen.

Measurements of spleen somatic-index were performed using naïve fish that were not part of experimental challenges. Spleen and body weights were determined for 5,645 individual fish from a total of 251 families (Table S2 in File S1, and Data S1 in File S2). Average family body weights used for genetic correlation and heritability estimates ranged between 4 and 347 g (n = 5,188). Total body weight was determined using a PG5002-S DeltaRange balance (Mettler Toledo) to 0.01 g, or for larger fish, weighed with a Champ CW11 digital stainless steel balance (Ohaus) to 5 g. The spleen was then removed and care taken to completely cut away any attached fat and connective tissue. Spleens were weighed with an AB104 balance (Mettler Toledo) to 0.0001 g. Spleen somatic-index was calculated by dividing organ weight (mg) by total body weight (g).

### Odd-year Rainbow Trout Pedigree, Mating Structure and Phenotyping

BCWD survival evaluation was initiated in 2005 (G_0_ population) using 71 full-sib (FS) families from the 2005 year class that have been described [10]. Four families with high survival (denoted R for resistant) and four families with low survival (denoted S for susceptible) were identified for further study [11]. The non-disease challenged siblings from six of the eight R and S families were used as breeders to generate the F_1_ QTL (2007) mapping population.

In 2007, two groups were phenotyped for BCWD resistance and spleen size. The first population G_1_ (2007) consisted of 97 FS families, of which 63 were part of the 2007 select line [9], and 34 were additional families created by outcrossing a 2005 select-line parent with one that was not. The second group, F_1_ QTL (2007), was created to detect QTL for BCWD resistance and consisted of 15 FS families from RxR, RxS and SxS parents [16]. Five of the RxR families were also evaluated in the G_1_ (2007) challenge. Spleen size was measured in both the G_1_ and F_1_ BCWD QTL (2007) families (n = 107 FS families) (Table S2 in File S1).

In 2009, three populations were phenotyped in several challenge experiments. The first experiment included 10 families from an F_2_ QTL#1 mapping experiment (challenge #5, Table S1 in File S1) designed to detect BCWD QTL [16]. The second experiment included 114 families G_2_ (2009) of which 96 families were from the select line [9], as well as 18 susceptible families derived from the same base population G_0_ (2005) as the select line (challenge #6, Table S1 in File S1). The BCWD select line has since been designated as ARS-Fp-R and the susceptible line as ARS-Fp-S and both lines have been maintained as closed populations [12]. The third experiment (challenge #7, Table S1 in File S1) included a total of 8 families that were part of F_2_ BCWD QTL mapping experiments, QTL#2a and 2b (2009), as well testing 5 families of the ARS-Fp-R line and 5 families from the ARS-Fp-S line that were part of a previously described field trial validation study [12]. The 10 families from the ARS-Fp-R and ARS-Fp-S lines were also represented in G_2_ (2009) challenge experiment.

### Even-year Rainbow Trout Pedigree, Mating Structure and Phenotyping

Families used in this study from the even-year line were a synthetic population developed from the crossing of six domesticated strains (listed in descending order of % contribution): 1) University of Washington, Donaldson; 2) Kamloops/Puget Sound Steelhead cross; 3) Ennis National Fish Hatchery, Arlee; 4) Ennis National Fish Hatchery, Shasta; 5) Idaho Department of Fish and Game, Hayspur; and 6) Kamloops. Phenotyping of 100 full-sib families from the even-year line was initiated in 2006 and has been described previously [11].

In 2008, a total of 15 full-sib families were created to detect QTL that influence spleen size, using parents that were from families evaluated in 2006 for SI, and categorized as large spleen size (H for high SI) and small spleen size (L for low SI). Five of each HxH, HxL and LxL F_1_ SI QTL (2008) families were created and offspring assayed for both post-challenge survival (n = 120 fish per family, mean age 114 days) and SI in naïve fish measured at five intervals over a 500-day growth period to determine the stability of this phenotype (mean post hatch age ± SD of the five intervals were 145±7, 170±6, 241±7, 323±6, and 633±5 days with mean BW ranging from 6 g to 3,716 g; Table S2 in File S1).

### Estimation of Heritability and Genetic Correlation

Heritability of survival following BCWD challenge (binary phenotype; fish that died were assigned a value of 0 and fish that survived were assigned a value of 1) and SI of naïve fish, and the genetic correlation between the two traits, were estimated with single and 2-trait linear animal models using MTDFREML [17]. The model for survival following BCWD challenge included fixed effects of family mean body weight at the time of challenge (linear covariate) and challenge date and a random effect of challenge tank nested within family. The effect of challenge tank is completely confounded with family, and thus accounts for non-additive genetic effects as well as common environmental effects shared by members of a full-sib family. The model for SI included fixed effects of age at the time of measurement (linear covariate) and year and a random effect of family. The environmental covariance between the two traits was fixed at 0 because only one phenotype was measured per fish and there is no known or postulated environmental correlation. Variance of the likelihood in the simplex less than 1×10^−9^ was used as the convergence criterion. Global minimum was assumed when −2×logarithm of likelihood was identical to the third decimal place after “cold” restarts of the simplex with prior estimates of variance components. The even- and odd-year populations differed in genetic background, and thus the datasets were analyzed separately. A total of 4,472 and 26,828 animals were used in the calculation of the inverse of the numerator relationship matrix for the even- and odd-year datasets, respectively. While all data from disease challenge studies were included, we excluded fish on which spleen size measurements were made that were larger than 347 g from the calculation of genetic correlation in order to reduce the possibility that we were measuring different traits in adult vs immature fish. Standard errors of the genetic correlation estimates were approximated as [(1– r^2^_A_)/√2]×√[(σ_h_2_x_ • σ_h_2_y_)/(h^2^_x_ • h^2^_y_)], where r^2^_A_ is genetic correlation (squared), h^2^ is heritability, and σ_h_2 is standard error of the heritability estimate [18].

### Complex Segregation Analysis of Spleen Index

In order to examine the mode of inheritance and identify potential families for QTL mapping, complex segregation analyses (CSA) were carried out using phenotypic data collected from even-year line fish. In this analysis, Mendelian transmissions of a single gene locus are simultaneously estimated with the patterns of covariance expected in polygenic inheritance [16], [19]. Records from 1,348 fish were used in these analyses and included 1,048 fish from fifteen F_1_ SI QTL (2008) families and 300 records from full sibs of the parents (2006 year class). Total body weight, age and year were subjected to multivariable regression analyses using STEPWISE model selection with SAS Proc REG (SAS, 2007) to identify fixed effects and covariates to include in the Bayesian segregation analyses (BSA) models. The BSA was carried out using computer program iBay [20] and eight mixed-inheritance models were evaluated and compared as described previously [16].

### Identification of a Family for SI and BCWD Linkage Analyses

A single family, designated 2008132, was selected for linkage analyses based on both the visual inspection of SI phenotype distribution and predicted major segregating locus or loci from BSA (Table S3 in File S1). Over a 100-day growth period, a total of 327 fish from family 2008132 were phenotyped for SI and fin-clipped (Data S2 in File S2). Selective genotyping was performed using 196 fish from the SI distribution: 89 fish had SI<1.22 (27.2% of fish sampled), 103 fish had SI>1.45 (31.5% of fish sampled), and 4 fish were sampled from the middle of the distribution.

To determine whether SI QTL also co-localized with BCWD resistance QTL, family 2008132 was further utilized in a BCWD linkage analysis. A total of 120 fish were subjected to BCWD challenge at 114 days post-hatch and averaged 5.0 g mean body weight. Three traits were measured in the challenge: 1) percent survival (Survival status), 2) day of death including survivors (Survival days), and 3) presence of a lesion at the injection site (Disease lesion) (Data S3 in File S2). Forty-four fish died (37%, n = 120 total) by day 21 with a mean day of death of 8.2 days. Genotyping was performed on 96 fish that included all mortalities and a random sample of 52 of the 76 survivors.

For the initial SI genome scan, a total of 336 microsatellite markers were genotyped using standard methods [21]. Once, SI QTL were identified, a BCWD QTL chromosome scan was targeted to eight chromosomes (Omy1,3,5,10,13,16,and 19) that were initially identified as containing genome-wide or suggestive SI QTL, and a total of 77 microsatellite markers were used to genotype BCWD challenged fish. Finally, a combined map was built from both SI and BCWD phenotyping/genotyping data sets that included 307 out of the total 341 markers used for genotyping (Data S4 in File S2). All markers had been previously mapped to the NCCCWA genetic map [21], [22].

### Testing Loci for Mendelian Segregation Distortion

All microsatellite loci were tested for Mendelian segregation distortion (MSD). In outbred rainbow trout FS families such as those used here, the progeny of an informative QTL mapping family can have any of these marker genotype proportions: 1∶1; 1∶2:1 and 1∶1:1∶1. Within individual FS families, the marker genotype counts were performed using a customized Perl script (available from the authors upon request). Chi-Square goodness-of-fit test of marker genotype counts were compared to expected proportions under Mendelian segregation using SAS Procedure FREQ [23] set to a default significance level of α = 0.01. The Monte Carlo (MC) estimation of exact *P-*values was estimated using 100,000 permutations. Marker loci with significant MSD were not used in the QTL analysis unless these loci had high quality marker genotype data (i.e., minimum genotyping errors).

### QTL Analysis

Before QTL analysis, we performed STEPWISE model selection with SAS Proc REG [23] to assess the contribution of the covariate age to the predictive power of response variable models (spleen related traits). The covariate age had significant effect on body weight, spleen weight and SI (*P*<0.05); and the effect of the covariate age on *ln*(SI) was close to the significance level (*P* = 0.0507). Subsequently, we decided to include the covariate age in the linear model used in the QTL analysis to minimize the variance in the sampled population. Basic statistical measures for the response variables were calculated using SAS Proc UNIVARIATE [23] (Table S4 in File S1).

The QTL genome scans were performed using a sire half-sib (HS) and a dam HS family analysis, separately, with the HS regression analysis module from software GridQTL [24]. The software GridQTL implements a multi-marker approach of interval mapping in HS families as described by Knott et al. [25]. This method of QTL analysis does not assume the parents had fixed QTL alleles, instead it relaxes the assumption of fixed QTL allele [25]. A QTL with a gene substitution effect is fitted at 1-cM intervals along the chromosome using this one-QTL model,
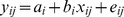
Where *y_j_* is phenotype of individual *j* from sire or dam *i; a_i_* is average effect for HS family *i; b_i_* is regression coefficient within HS family *i* (substitution effect of QTL); *x_ij_* is conditional probability for individual *j* within HS family *i* of inheriting allele 1 (or 2); and *e_ij_* is the residual error.

In the HS regression analysis, the likelihood ratio (LR) test statistic was defined as 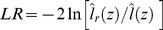 where *ln* stands for natural logarithm, 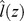 is the likelihood function evaluated at the maximum likelihood estimate (MLE) for the full model that includes polygenic and QTL effects, and 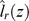 is the MLE for the restricted model under which *r* parameters of the full model are assigned fixed values [26]. The *P*-value was calculated assuming an *F*-value distributed with numerator DF equal to the number of sires or dams, and denominator DF equal to the total number of offspring minus twice the number of sires or dams [25]. The chromosome-wide *F*-value 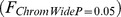 and experiment-wide *F*-value 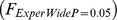 were estimated using 10,000 permutations with software GridQTL [24]. QTL with *F*-value ≥ *F_ChromWide P = 0.05_* were defined as suggestive QTL (*) and QTL with *F*-value ≥ *F_ExperWide P = 0.05_* were defined as significant QTL (**). The proportion of phenotypic variance explained by the QTL was calculated as 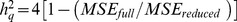 where *MSE_full_* and *MSE_reduced_* are the mean squared error of the full and reduced model, respectively [25]. The estimated 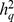 was corrected for selective genotyping. The 95% QTL confidence intervals (CI95) were estimated using 10,000 bootstraps with re-sampling with software GridQTL [24].

The QTL analysis proceeded in three steps. First, all chromosomes were genome scanned for QTL affecting the analyzed trait performing regression interval mapping using one-QTL model. At this step: (i) the chromosome-wide significance threshold level 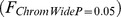 to declare suggestive QTL was determined using 10,000 permutations; and (ii) the CI95 for each suggestive QTL (and above) was determined using 10,000 bootstraps with re-sampling. Second, the chromosomes with QTL detected at the 5% chromosome-wide significance level (and above) were re-scanned for additional QTL using one-QTL model and including in the model the effect of already detected QTL until not detecting more QTL in a chromosome. By accounting the already detected QTL as background effects, the variance caused by them is removed, thus potentially revealing previously undetected QTL. Third, the chromosomes with two detected QTL were re-scanned fitting two-QTL models. In chromosomes with two detected QTL: (i) the 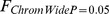 significance level for one QTL at a time was estimated while accounting for the effect of the other detected QTL using 10,000 permutations; and (ii) the CI95 for each QTL was determined by fixing alternatively the effect of each detected QTL using 10,000 bootstraps with re-sampling [27].

For QTL identified as significant or suggestive, the allele substitution effect for each parent was tested using a one-sided *t*-test (absolute *t*-value) with one DF. For this test, where the overall QTL effect had already been determined, a parent was defined as QTL heterozygous using a nominal 10% significance threshold. The QTL allele substitution effect was expressed in phenotypic standard deviation units (i.e., estimated effect size/SD of the phenotype).

### Data Availability

All the significant and suggestive QTL identified in this work for SI and for BCWD resistance were uploaded onto the rainbow trout QTL database within the Animal QTLdb (http://www.animalgenome.org/QTLdb) [28].

## Results

### Spleen Index Heritability and Genetic Correlation with BCWD Survival

If naïve spleen size and BCWD resistance are influenced by tightly linked or common genes, we hypothesized there should be a positive genetic correlation between the two traits. To test this hypothesis, two breeding populations of pedigreed rainbow trout, from 5 year classes, were phenotyped for both traits. The two populations have different founder contributions, and thus genetic background, and were thus analyzed separately. Heritability estimates for survival following BCWD challenge were 0.23±0.09 (± s.e.) in the even-year population and 0.25±0.03 in the odd-year population. Heritability estimates for SI were 0.56±0.18 (± s.e.) in the even-year population and 0.60±0.15 in the odd-year population. Estimates of genetic correlation between family SI and family BCWD survival were 0.45±0.20 in the even-year population and 0.16±0.12 in the odd-year population. The significance of these estimates were formally tested for difference from zero using a two-tailed t-test (comparison of models: *r_g_* unconstrained vs *r_g_* fixed at zero, *P* = 0.03 even-year population and *P* = 0.19 odd-year population). The even- and odd-year genetic correlations significantly differed (P = 0.022). In summary, these data indicate a significant and positive genetic correlation in the even-year line and a weak and non-significant genetic correlation in the odd-year line. Variation in genetic correlation across populations is not unusual as these estimates have large associated errors that may reflect sampling bias or differing population gene frequencies (18).

### Mode of SI Inheritance

**Figure 1:**
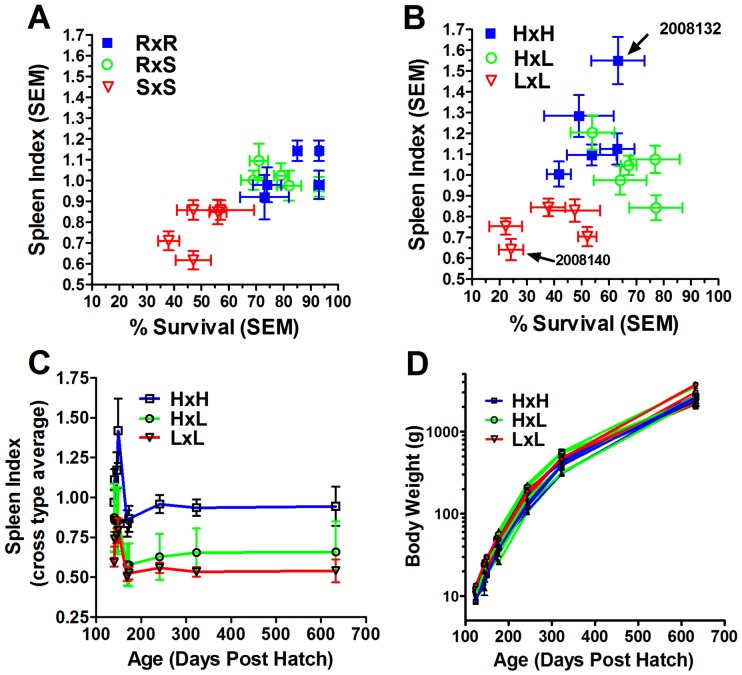
Family phenotypic correlation between naïve-animal spleen index (SI) and survival following challenge with *F. psychrophilum*. (A) Trait correlation in fifteen 2007 year-class families selected based on BCWD resistance phenotype. Families were created using parents whose full-sibs were BCWD challenged in 2005 and identified as highly disease “resistant” (R) or “susceptible” (S) families. Five RxR (blue), RxS (green) and SxS (red) crosses were assayed for both survival (n = 200 fish per family, mean age 118 days,) and SI (n = 20 per family, mean age 128 days). (B) Trait correlation in 2008 year-class families. Fifteen families were created from fish whose full-sibs were evaluated in 2006 for SI and categorized as high SI (H) and low SI (L). Five HxH (blue), HxL (green) and LxL (red) crosses assayed for both survival (n = 120 fish per family, mean age 114 days,) and SI (n = 20 per family, mean age 145 days). (C) Stability of SI in HxH, HxL and LxL cross types. Values represent an average (±1 SEM) of 5 families per cross type. (D) Equivalent growth rates of the F_1_ SI QTL (2008) families.

Our initial observation of a positive phenotypic correlation between SI and BCWD resistance in the 2005 and 2006 year classes led us to further investigate the mode of inheritance and genetic architecture of spleen size. A total of 15 families were created in 2007 and 2008 by mating animals which were highly divergent in either their BCWD survival or SI phenotypes. We observed a positive phenotypic correlation *r* = 0.80 between both traits across families in the 2007 F_1_ BCWD QTL population (Figure 1A). Both SI and survival were similar between RxS and RxR crosses suggesting that the pattern of inheritance of these traits may be co-dominant or dominant. In the 2008 F_1_ SI QTL crosses, there was a positive phenotypic correlation between the two traits, *r* = 0.46 (Figure 1B), albeit lower than the phenotypic correlation observed in F_1_ BCWD QTL population. Furthermore, there was a greater phenotypic variation in average SI between families which might be expected as these families were initially selected solely based on SI. Some HxL families survived BCWD challenge at a higher percentage than HxH crosses while SI of the HxL crosses was similar or slightly lower than HxH crosses. These phenotypic data suggest that the pattern of spleen inheritance may be additive or dominant in these families.

### Stability of SI during Growth

We previously observed that spleen size was a stable trait over a 50-day period during which fish weight increased 74% [11]. Given the high heritability of spleen size, we examined whether relative family spleen size would be stable over the entire growth cycle. Average family SI, apart from the initial measurements, was generally stable over time (Figure 1C), and growth of the 15 families was similar over a 500-day period (Figure 1D). Correlations among family means for SI across the five intervals ranged from 0.48 (*P*-value = 0.08; 95% CI = −0.07 to 0.81) to 0.86 (*P*-value <0.001; 95% CI = 0.63 to 0.96) and generally decreased as time between measurements increased (Table 1). This pattern of correlation was less consistent at the oldest age, which may be partly attributable to smaller sampling sizes at that age (n = 81 animals, range = 0 to 12 animals per family) and mean family body weights up to 3.7 kg (Table S2 in File S1).

**Table 1:** Correlations among family mean SI.

mean age^1^, d	170	241	323	633
145	0.86	0.82	0.70	0.71
170		0.83	0.76	0.76
241			0.88	0.60
323				0.48

1 Days post-hatching.

### Genetic Architecture of Spleen Size

We next examined whether SI was influenced by the action of a segregating locus of large effect. For these studies, we focused on the even-year line because we measured a 2.8-fold greater genetic correlation between SI and disease resistance in this population. Polygenic and mixed inheritance models were compared using complex segregation analyses utilizing the F_1_ SI QTL (2008) dataset. Estimated marginal posterior means for variance components (data not shown) and major gene parameters of *ln*(SI) indicated that Model 7 (Dominant *A_1_*) had the highest Bayes factor (1,495) (Table 2). These results suggest dominant inheritance of at least one major gene segregating in the even-year spawning population.

**Table 2:** Estimated marginal posterior mean for variance components and major gene parameters of *ln*(SI) using polygenic and mixed inheritance models in Bayesian segregation analysis^1^.

Model^2^	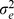	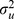	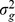	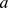	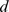	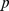	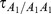	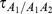	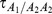	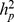	*log_e_ [p(y/H_i_)]* 3	Model tested	BF(*H_2_; H_1_*)^4^
1. General	0.011	0.110	3.408	1.36	−1.21	0.58	0.85	0.46	0.15	0.90	1,378.0	1 vs. 2	487.1
2. General fixed	0.087	[0]	35.834	5.72	−5.99	0.73	0.99	0.28	0.02	[0]	890.8	2 vs. 3	315.3
3. Sporadic	0.156	[0]	[0]	[0]	[0]	[0.0]	[0.0]	[0.0]	[0.0]	[0]	575.5	4 vs. 3	715.4
4. Polygenic	0.017	0.131	[0]	[0]	[0]	[0.0]	[0.0]	[0.0]	[0.0]	0.88	1,290.9	7 vs. 5	140.4
5. Dominant *A_2_*	0.015	0.121	0.055	0.23	0.23	0.45	[1.0]	[0.5]	[0.0]	0.89	1,354.3	5 vs. 1	−23.7
6. Additive	0.015	0.107	0.035	0.29	[0]	0.55	[1.0]	[0.5]	[0.0]	0.87	1,337.6	6 vs. 1	−40.4
7. Dominant *A_1_*	0.010	0.110	0.031	0.20	−0.20	0.52	[1.0]	[0.5]	[0.0]	0.91	1,494.7	7 vs. 1	116.8
8. Codominant	0.012	0.115	0.405	1.18	−0.13	0.46	[1.0]	[0.5]	[0.0]	0.90	1,343.2	8 vs. 1	−34.8

1 Bayesian segregation analysis of *ln*(SI) performed with software iBay version 1.46 [20]. The Gibbs sampler had these characteristics: number of iterations per chain = 1,200,000; burn-in period = 600,000; thinning = 10,000; collected samples per chain = 60; total chains = 20; and total collected samples = 1,200.

2 Model parameters: error variance 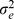; polygenic variance 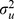; major gene variance 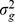; major gene additive effect 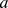; major gene dominance effect 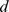; 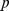
is frequency of spleen size-decreasing allele *A_1_*; 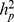 is the polygenic model heritability 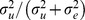; and transmission probabilities 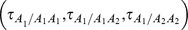 defined as the probability that a parent with any of the three genotypes 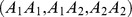 transmits the allele 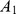 to its offspring.

3 *log*_e_ of the marginal density under the fitted model *H_i_*.

4 Bayes factor BF(*H_2_; H_1_*) = *p(y/H_2_)/p(y/H_1_)* is the ratio of the marginal likelihood under one model to the marginal likelihood under a second model, and *H_1_* and *H_2_* are the two competing models.

5 Value between squared brackets indicates the parameter was fixed to the value shown.

**Figure 2:**
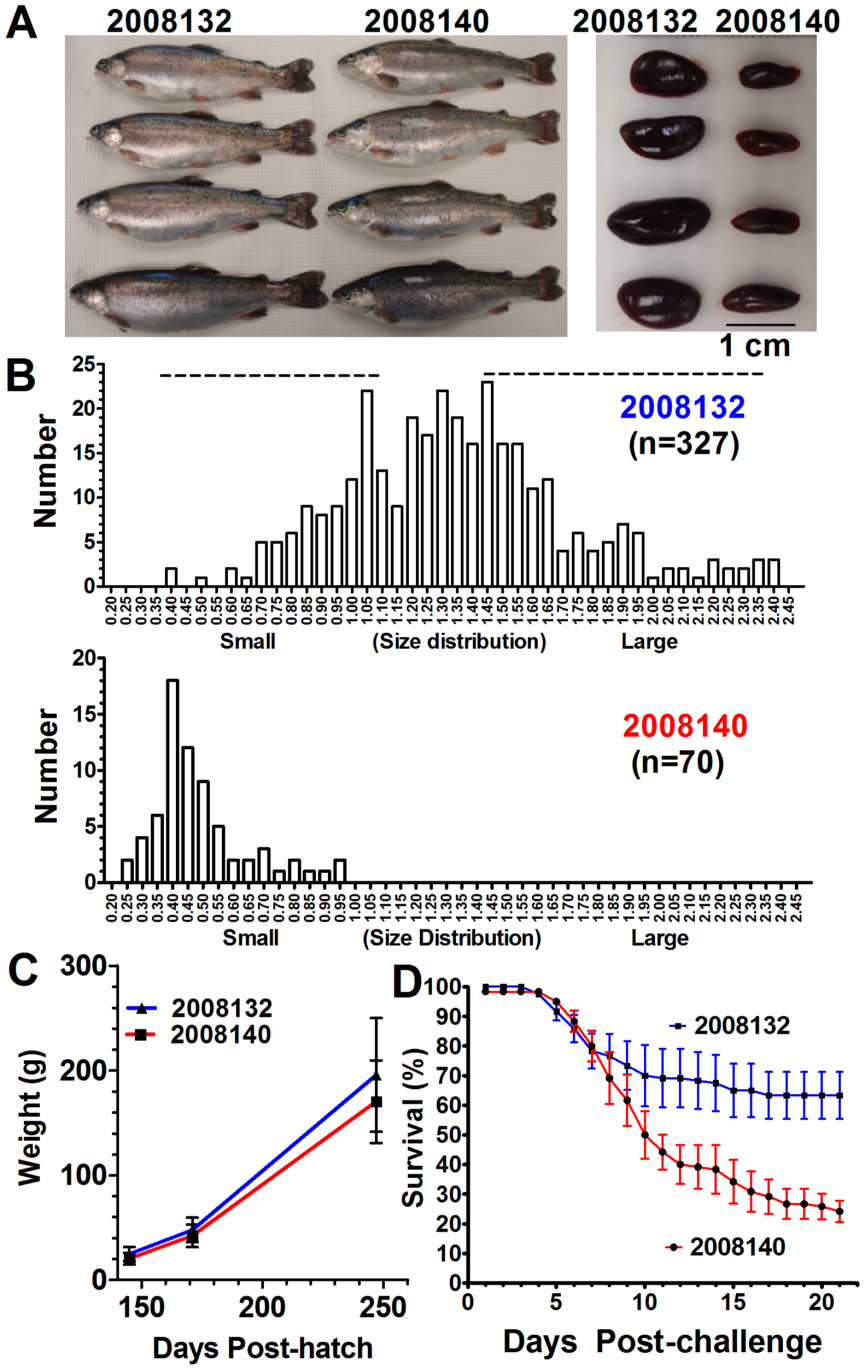
Rainbow trout families with divergent spleen indices: family with high average SI, 2008132, and low average SI, 2008140. (A) Four randomly selected fish and respective spleens. (B) SI phenotype distribution of families 2008132 and 2008140. (C) Average body weights (not statistically different). (D) Survival following laboratory injection challenge with *F. psychrophilum* (*P*<0.001, results are an average from triplicate tanks, n = 40 fish per tank).

The models assumed a single locus with autosomal Mendelian inheritance, and since the validity of this assumption was likely violated by the presence of multiple contributing loci and potential epistatic effects, we directly searched for the presence of QTL within a single family using a panel of microsatellite markers distributed across the rainbow trout genome. Based on modeling of Mendelian transmission probabilities estimated from the marginal posterior distributions after BSA (data not shown), we predicted single-locus genotypes for parents of 15 rainbow trout families (Table S3 in File S1). From these families, a single putative backcross mapping family, ID number 2008132, was chosen for QTL genome scan. This family was highly divergent in SI (Figure 1B and Figure 2A and B) and was derived from two high SI parents. Family 2008140 is included for comparative purposes and was created by mating two low SI parents (Figure 1B and Figure 2A and B). Growth of these families were similar (Figure 2C) while BCWD resistance differed significantly (Figure 2D).

**Figure 3:**
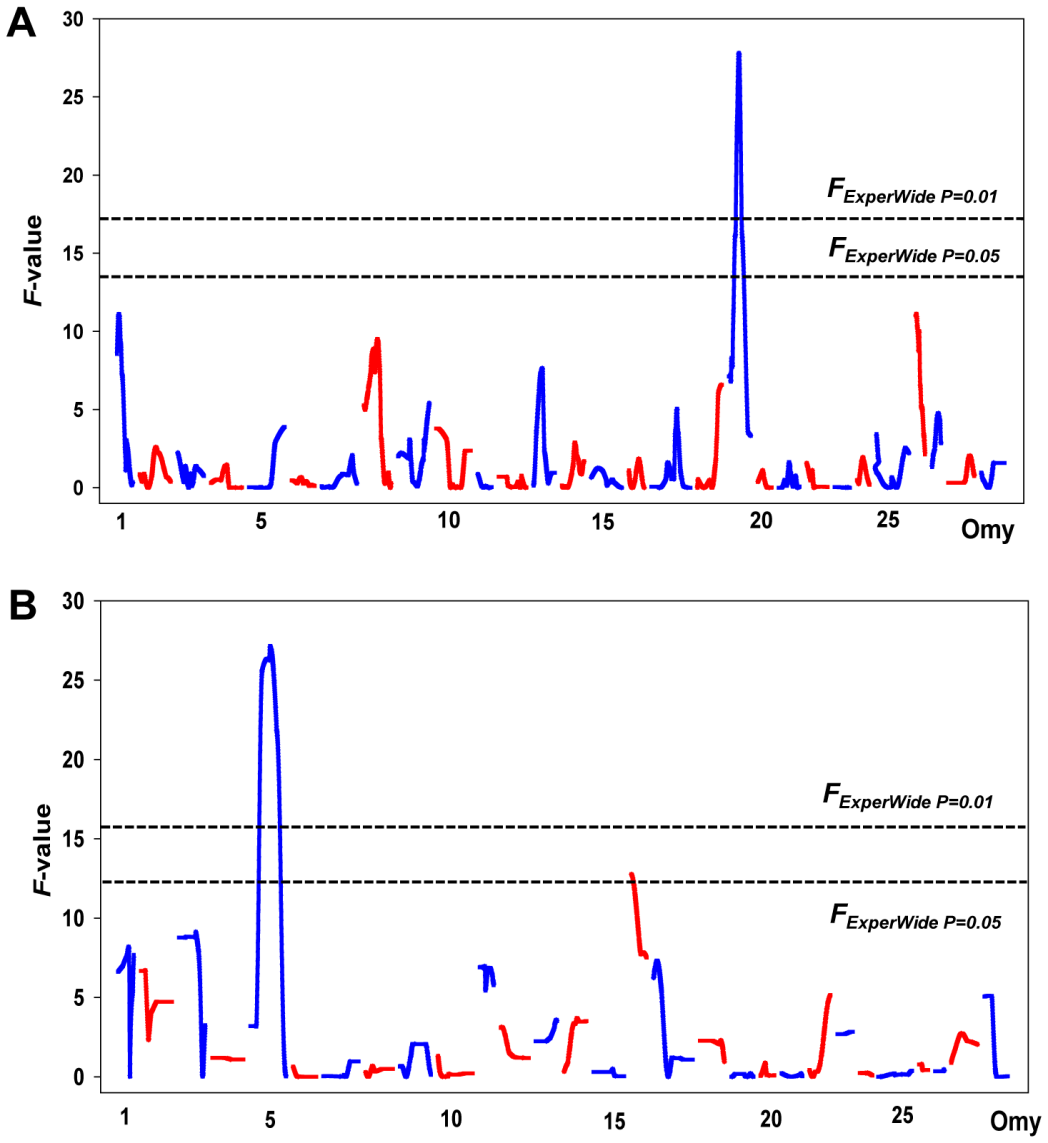
Quantitative trait loci for SI identified by half-sib (HS) family regression interval mapping in rainbow trout family 2008132. (A) Plot of QTL for dam alleles showing all chromosomes. A major QTL was identified on Omy19. (B) Plots of quantitative trait loci for sire allels showing all chromosomes. Major QTL were identified on Omy5 and Omy16.

The final *de novo* built genetic maps contained a combined 307 informative markers (Table S5 in File S1) with an average marker density of 6.5 cM, and was in agreement with the NCCCWA reference genetic map [29]. The analysis of SI and logSI using half-sib family regression, identified three significant QTL (*F* ≥ *F_ExperWide P = 0.05_*) influencing spleen size on chromosomes Omy5, 16 and 19 (Figure 3 and Table 3). The QTL on chromosomes Omy5 and Omy19 each accounted for 18% of the family phenotypic variance in spleen size. A total of 10 suggestive QTL (*F* ≥ *F_ChromWide P = 0.05_*) that accounted for between 4 and 7% of the phenotypic variance were identified on Omy1, 2, 3, 8, 11, 13, 17, and 18, and 26 (Table 3). The SI QTL were not confounded by body weight, as a significant QTL for body weight mapped only to Omy10 (*F_ExperWide_*<0.05) in addition to three suggestive QTL that mapped to Omy10, 22 and 29 (Table S6 in File S1).

**Table 3:** Significant and suggestive QTL for SI using half-sib family regression analysis^1^ in rainbow trout family 2008132.

					*F_ChromWide_*	*F_ExperWide_*			Closest marker^10^	
Category	Omy^2^	cM^3^	LR^4^	*F*- value^5^	*_P = 0.05_* 6	*_P = 0.05_* 7	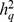 8	95% C.I.^9^	Left	Right
***Dam HS family***	1	5	10.76	11.09*	7.12	13.64	0.07	0.0–27.0	Ssa20_19NUIG	OMM1205
	8	39	9.27	9.52*	7.02	13.64	0.06	19.0–71.0	OMM1579	OMM1055
	13	25	7.46	7.63*	6.63	13.64	0.05	0.0–60.0	OMM1330a	OMY1UoG
	18	70	6.47	6.59*	6.36	13.64	0.04	2.0–70.0	OMM1303	OMM1352
	19	29	25.91	27.81**	6.65	13.64	0.18	16.0–34.0	OMM5216	OMM5327
	26	1	10.78	11.12*	5.97	13.64	0.07	0.0–22.0	OMY1189b	OMM5231a
***Sire HS family***	1	30	8.01	8.02*	5.97	12.28	0.05	4.0–45.0	OMM1152	OMM1355
	2	10	6.54	6.67*	4.93	12.28	0.04	4.0–81.0	OMM1391a	OMM1131
	3	48	8.89	9.12*	5.54	12.28	0.06	4.0–75.0	OMM4025	OMM1080
	5	57	25.31	27.12**	6.08	12.28	0.18	29.0–80.0	OMM1439	OMM5213
	11	4	6.78	6.92*	4.45	12.28	0.04	0.0–40.0	OMM3093	OMM1433
	16	0	12.33	12.77**	4.62	12.28	0.09	0.0–42.0	OMM1221	OMYRGT6TUFa
	17	10	7.17	7.32*	6.07	12.28	0.05	0.0–58.0	OMM1306	OMM1607

1 Half-sib regression interval mapping was performed with web-based software GridQTL [24].

2 Rainbow trout chromosome number [21], [22].

3 The chromosome location with the highest estimated *F*-value was defined as the most likely QTL location. The map cM units are based on genetic maps developed for this QTL genome scan study.

4 The likelihood ratio test statistic was estimated as 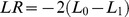 where *L_0_* is the log likelihood of polygenic model and *L_1_* is the log likelihood of the QTL model at the tested location (cM).

5 Asymptotically *F-*test statistic with the degrees of freedom(DF) being the number of sires or dams included for the numerator, and the total number of offspring minus twice the number of sires or dams for the denominator [25]. The QTL with *F*-value ≥ *F_ChromWide P = 0.05_* was defined as suggestive QTL (*) and QTL with *F*-value ≥ *F_ExperWide P = 0.05_* was defined as significant QTL (**).

6 Chromosome-wide *F*-value at *P* = 0.05 was estimated using 10,000 permutations with GridQTL [24].

7 Experiment-wide *F_ExperWide P = 0.05_* was estimated using 10,000 bootstraps with re-sampling with GridQTL [24].

8 The proportion of phenotypic variance explained by the QTL was calculated as 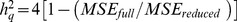 where *MSE_full_* and *MSE_reduced_* are the mean squared error of the full and reduced model, respectively [25]. The estimated was corrected for selective genotyping according to Darvasi and Soller [61].

9 The 95% confidence intervals for each suggestive and significant QTL was determined using 10,000 bootstraps with re-sampling with software GridQTL [24].

10 The closest markers flanking (left and right) the chromosome location with the highest estimated *F*-value in the QTL scan.

In order to test the hypothesis that spleen size QTL co-localize with BCWD QTL, a separate scan of eight chromosomes was performed using 96 fish from family 2008132 (Figure 1D). A single significant QTL (*F* ≥ *F_ExperWide P = 0.05_*) was identified on Omy19 influencing survival status as well as survival days (Table 4). This QTL explained 57% and 67% of the family phenotypic variance in survival status and survival days, respectively. The QTL on Omy19 also explained 24% of the phenotypic variance in injection-site lesion prevalence. In addition to Omy19, a suggestive QTL was identified on Omy3 influencing survival status that explained 16% of the phenotypic variation. Since both Omy19 and Omy3 contained QTL for both traits we next examined whether the phenotypes associated with individual marker alleles were correlated. In other words, individuals having an allele associated with increased resistance should also have a higher SI as compared to full-sibs lacking the allele. Six individual QTL marker alleles on Omy19 (BX873975a through OMM1412b) were associated with a 0.12–0.21 increase in SI units and these fish also exhibited a 20–39% increase in BCWD survival and 3.2–6.5 increased days until death (Table 5). Thus, while the QTL peaks for SI and BCWD resistance on Omy19 were 26 cM apart, the 95% CI overlapped and the allele effects were correlated and thus likely contribute to the observed genetic correlation between the two traits in the even-year line. In contrast to Omy19, the effects of the suggestive QTL on Omy3 were opposite: fish with alleles corresponding to modestly higher SI had a lower survival rate (data not shown). In summary, the coincident linkage of QTL affecting both SI and BCWD traits on Omy19 supports the hypothesis of colocalizing (closely linked) loci affecting both phenotypes. Given the low resolution of our genetic maps, our data do not exclude the possibility of a single pleiotropic locus in family 2008132.

**Table 4:** Significant and suggestive QTL for BCWD traits using half-sib family regression analysis^1^ in rainbow trout family 2008132.

						*F_ChromWide_*	*F_ExperWide_*			Closest marker^10^	
Category	Trait	Omy^2^	cM^3^	LR^4^	*F*-value^5^	*_P = 0.05_* ^6^	*_P = 0.05_* ^7^	^8^	95% C.I.^9^	Left	Right
***Dam HS family***	Survival days	19	3.0	18.06	20.02**	7.04	11.91	0.67	0.0–38.0	OMM1125	BX873975a
	Survival status	19	3.0	15.39	16.82**	7.00	11.96	0.57	0.0–47.0	OMM1125	BX873975a
	Disease lesion	19	5.0	6.83	7.13*	6.76	11.50	0.24	1.0–55.0	BX873975a	OMM1124a
***Sire HS family***	Survival status	3	9.0	4.84	5.00*	4.66	10.40	0.16	3.0–48.0	OMM1016a	OMM5121

1 Footnotes are the same as for Table 3.

**Table 5:** Association of alleles with spleen size and BCWD survival traits.

							BCWD Resistance Traits				
			Dam alleles assoc. with		Spleen Index		Survival status		Survival days		
Omy	cM	Marker	Increase	Decrease	(SI Units^1^)	*P> t*	(Survival %^1^)	*P> t*	(Days^1^)	*P> t*	
19	3.3	BX873975a	207	Null^2^	0.12	0.0120	20%	0.0030	3.2	0.0014	
19	6.2	OMM1124a	161	199	0.19	0.0070	39%	0.0002	6.5	<.0001	
19	7.5	OMY120INRAb	192	298	0.21	0.0025	32%	0.0037	5.2	0.0008	
19	9.5	OMM1657	238	234	0.18	0.0101	31%	0.0027	5.1	0.0007	
19	10.6	OMM1134a	160	152	0.19	0.0062	27%	0.0100	4.5	0.0030	
19	10.7	OMM1412b	314	340	0.17	0.0120	22%	0.0449	3.7	0.0194	
19	13.5	OMM1440	161	249	0.14	0.0069		n.s.^3^		n.s.	
19	19.4	OMM1739	168	171	0.27	0.0001		n.s.		n.s.	
19	23.6	OMM5216	132	134	0.33	<.0001		n.s		n.s.	
19	37.1	OMM5327	170	204	0.26	0.0001		n.s.		n.s.	

1 Allele effect is estimated by regression of SI, survival days, or survival status with the dam allele. The two alleles from the dam are listed, and for convenience, the effect on phenotype is listed in positive units.

2 Null allele.

3 n.s. indicates non-significant test (*P*>0.05).

## Discussion

Breeding for disease resistance is being increasingly utilized in salmonid aquaculture to improve fish survival and welfare [30], [31], [32], [33]. Previously, we have shown that BCWD resistance in rainbow trout is a moderately heritable trait that responds to selection [9]. Furthermore, we have recently demonstrated, in five field trials completed to date, a higher on-farm survival of the ARS-Fp-R line following natural BCWD epizootics as compared to either control lines or hatchery stocks ([12], Wiens G. unpublished data). Thus, the genetic control of BCWD disease resistance and correlated traits is of interest for both practical advancement of our breeding objective as well as for understanding biological processes involved in the host response to bacterial pathogen challenge. The contribution of the spleen to anti-bacterial immunity is poorly understood in lower vertebrates and factors influencing spleen size and functions in lower vertebrates have received little attention (reviewed in [34], [35], [36]). We previously postulated that since SI is an easy-to-measure quantitative trait it might be useful to dissect the genetic mechanism(s) of disease resistance and/or possibly follow the introgression of resistance alleles into other commercial rainbow trout stocks [11]. Herein, we report that rainbow trout spleen somatic-index is a moderately to highly heritable trait as measured under laboratory conditions and that there is a positive genetic correlation with specific resistance to BCWD in an even-year spawning population. The genetic correlation was weak and non-significant in the NCCCWA odd-year line. Estimates of genetic correlations are known to be subject to large sampling errors and are seldom precise (18). Furthermore, genetic correlations are strongly influenced by gene frequencies that may differ markedly between populations (18). Thus it is not surprising that there were differences between the odd- and even-year population estimates. Given that the magnitude of the genetic correlation varied between lines, we consider SI of unknown and probably limited value as a single selection criterion to develop BCWD resistant rainbow trout. Nevertheless, the identification of a major QTL on Omy19 in family 2008132 associated with both traits identified a region of closely linked loci, or possibly a locus with pleiotropic effect that will be the basis of further mechanistic study.

### Heritability of Spleen Size and Identification of QTL

To our knowledge, our estimates of spleen size heritability are the first reported for a teleost. The moderate to high heritability estimates of SI in rainbow trout is not unexpected as morphological traits are generally highly heritable, especially when measured under defined and optimal environmental conditions [37]. Fish measured in our study were reared under constant temperature, provided a standardized feeding regime and were from pathogen-free certified broodstock. It is likely that heritability estimates measured under aquaculture production conditions may produce lower estimates as a number of environmental factors are known to influence spleen size. These include pathogen exposure and sexual maturity [11], [38], [39]. Our estimates of heritability may also be upwardly biased by the mating structure utilized in our study. We currently monitor SI in our unexposed fish as well as in disease challenge studies as a general marker for immunological activation. This study provides a large dataset of naïve fish spleen and body weight measurements (n = 5,645 fish) obtained at ages 93 through 637 day post-hatch and thus is a baseline for future population comparison.

The positive genetic correlation of SI with disease resistance in the even-year spawning population led us to examine the genetic architecture of both traits. Here we modeled the inheritance of spleen size using complex segregation analyses and found evidence for dominant inheritance of small spleen size with at least one gene with major effect on the trait segregating within the even-year population. Our previous efforts to model BCWD resistance within our odd-year population using Bayesian methods of segregation analysis suggested that 6–10 QTL explain 83–89% of phenotypic variance and the sum of these effects may be due to co-dominant or dominant disease-resistant alleles plus polygenic effects [16]. In summary, the modeling of both traits, using only the phenotypic and pedigree data, suggested the presence of multiple QTL with complex inheritance in our trout population. Results from SI QTL genome-wide scans with microsatellite markers identified two highly significant SI QTL on Omy19 and 5 that each separately accounted for 18% of the phenotypic variance, as well as a third significant QTL on Omy16 that accounted for 9% of phenotypic variance in family 2008132. In addition, we identified ten suggestive QTL each contributing a minor amount to the SI phenotypic variance. The oligogenic nature of rainbow trout SI inheritance is not surprising as spleen weight in inbred lines of mice is influenced by at least 10–12 QTL [40], [41]. Most of the identified QTL from the murine studies were pleiotropic, correlating with other body size or organ weight measurements. The SI QTL we identified in our study, however, were not confounded by body size as we identified only one significant body weight QTL on Omy10 as well as three suggestive QTL, and none of these overlapped with SI QTL. The significant body weight QTL on Omy10 may have been previously identified in two other studies [42], [43]. In summary, several major and numerous moderate QTL affecting SI were identified in family 2008132.

### Mechanistic Connection between Spleen Size and Disease Resistance

**Figure 4:**
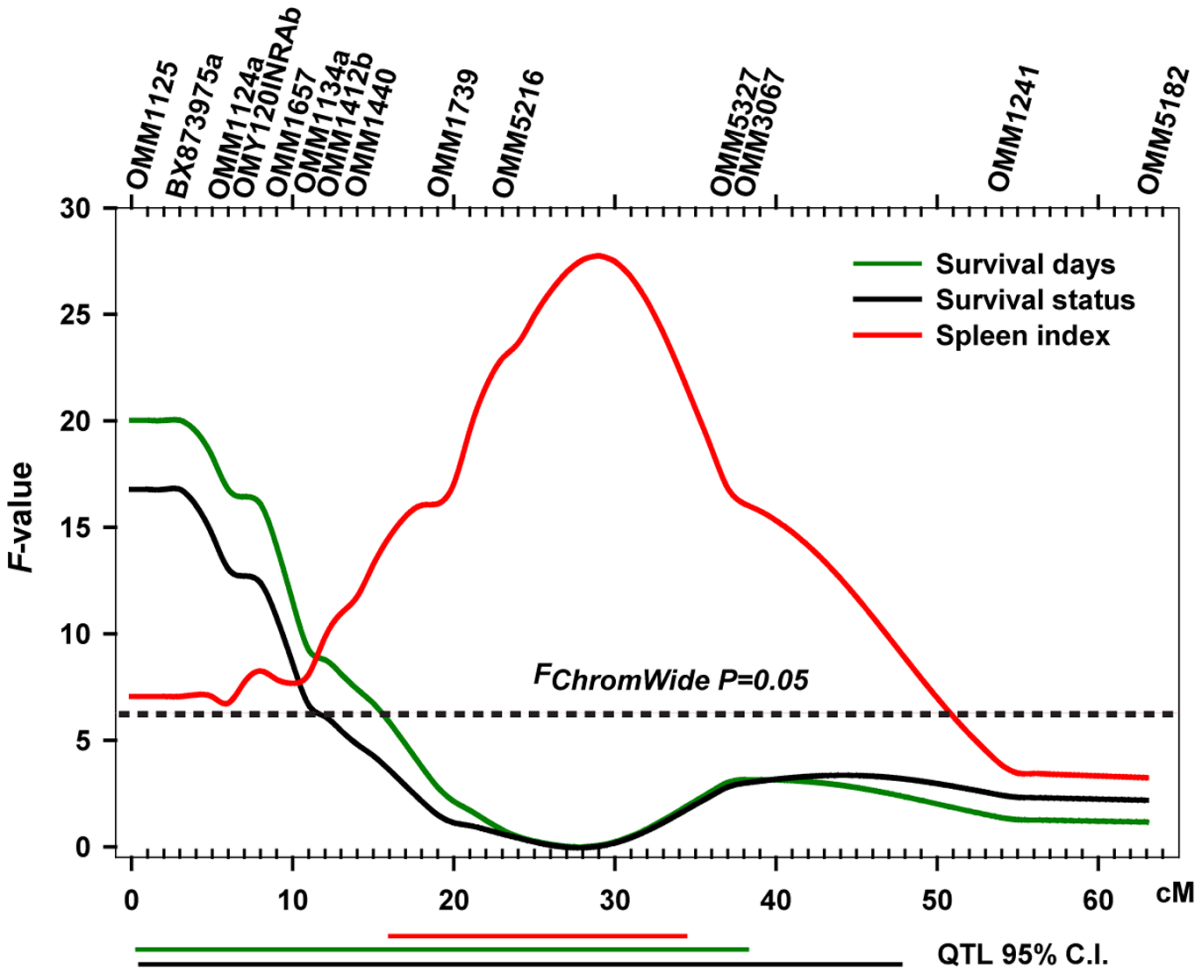
Plot of Omy19 showing QTL intervals for SI and BCWD resistance traits. The location and designation of microsattalite markers used to identify the QTL are listed above the figure. The 95% confidence interval for each QTL is indicated below the graph by an individual line for each trait. Data for both SI and BCWD QTL fit a single QTL model with no evidence for a second QTL.

Selective genotyping of BCWD susceptible and resistance fish from family 2008132 identified a major BCWD QTL on Omy19. This QTL accounted for 57% of the phenotypic variance of survival days and 67% of the survival status variation in family 2008132. We caution that although our analysis was adjusted for selective genotyping, these values may still be upwardly biased. A total of six markers (BX873975a-OMM1412b) were significantly associated with both traits (Figure 4 and Table 5) raising the hypothesis that the two QTL are in linkage disequilibrium. The peak signal for the SI QTL and BCWD QTL were separated by a distance of 26 cM which argues that the two QTL are most likely influenced by separate genes. However, we cannot exclude the possibility of a single locus with pleiotropic effect due to the imprecise nature of the genetic maps and the overlapping QTL 95% confidence intervals. Also, in a separate, three-generation study of multiple families from the NCCCWA odd-year breeding line, we have identified a family with an Omy19 BCWD QTL that overlaps with the BCWD QTL reported here, but the peak is located 14 cM closer to the SI QTL (peak signal 17 cM, 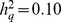) (Vallejo et al., unpublished data). The Vallejo et al. study also identified additional BCWD QTLs that were not detected in this study, demonstrating the genetic complexity of BCWD resistance in our two rainbow trout populations. We also point out that most of the detected SI QTLs did not co-localize with BCWD QTL. For example, the major SI QTL identified on Omy5 does not influence BCWD resistance and the inverse correlation between the two suggestive QTL on Omy3 may confound and dilute the population-genetic correlation between SI and BCWD. These results underscore the genetic complexity of each trait that likely contributes to the different estimated genetic correlation between odd- and even-year populations. To our knowledge, no immune genes have been reported to map to Omy19 [44], [45], [46], [47] and thus this chromosome will be a target for future fine mapping of both SI and BCWD QTL. In summary, our study provides evidence for a common genetic architecture on Omy19 explaining a component of the genetic correlation between SI and BCWD resistance. Given the limits in the resolution of our mapping of both traits, we cannot distinguish between linkage disequilibrium or other possible causes such as pleiotropy [48], [49]. Further efforts are currently underway to phenotype additional crosses made using full-sibs of family 2008132 and to genotype offspring using higher marker density on Omy19.

To our knowledge, this study represents the first report on using spleen size to identify a family demonstrated to be informative for BCWD resistance mapping. The identification of SI can be added to other indirect phenotypic traits that have been found to correlate with disease resistance. These include viral infectivity of fin explants, or fibroblastic cell lines from cloned trout, that correlate with the susceptibility of either the progeny or the adult fish stocks to viral haemorrhagic septicaemia virus [50], [51]. In Atlantic salmon, gill lesion score is correlated with survival results following experimental amoebic gill disease challenge or natural infection [52], [53]. At present, we have not identified a mechanistic connection between spleen size and BCWD resistance. The transcriptional response of BCWD resistant and susceptible rainbow trout homozygous clones have identified differential up-regulation of IL1 and IL10 gene transcript in susceptible clonal line B57 while complement factor C3 gene transcript showed stronger up-regulation in resistant clonal line A3 at day 5 post-challenge [54]. It will be of interest to determine whether these gene candidates are differentially expressed in our QTL population and whether they map to genomic regions with QTL detected in this study.

Recently, a number of major QTL influencing resistance to viral and parasitic diseases in rainbow trout and Atlantic salmon have been identified [55], [56], [57], [58], [59], [60] that may lead to specific selection markers. Identification of a more specific marker associated with BCWD disease resistance on Omy19 would add to this growing list and may have future utility to exploit both between- and within-family selection in aquaculture selective breeding.

## Conclusions

To our knowledge, this is the first report of a positive genetic correlation between spleen somatic-index and resistance to challenge with *F. psychrophilum* in a domesticated population of rainbow trout. Spleen index is a moderately to highly heritable trait as determined using two independent methods: 1) Bayesian segregation analysis using a mixed inheritance models (i.e. polygenic+major gene effects), and 2) maximum likelihood estimation using MTDFREML. We report here the first identification of a SI QTL on Omy19 that has coincident linkage with BCWD QTL. We suggest that our pedigreed rainbow trout lines may serve as a model system for mechanistic study of factors controlling spleen size and dissection of the genetic basis of resistance to BCWD.

## Supporting Information

File S1 **Tables S1–S6.** Rainbow trout year class, body weight, age and dose of challenge with *F. psychrophilum* strain CSF259-93 (Table S1). Measurement of spleen size in odd-year and even-year line rainbow trout populations (Table S2). Predicted major gene genotypes for parents of rainbow trout families after Bayesian segregation analysis^1^ of *ln*(spleen index) (Table S3). Basic statistical measures for spleen related traits in rainbow trout from cross 2008132 (Table S4). Genotyped and mapped microsatellite markers per rainbow trout chromosome (Table S5). Significant and suggestive QTL for body weight using half-sib family regression analysis in rainbow trout family 2008132 (Table S6).
(DOCX)

File S2 **Data S1–S4.** Complete dataset of BCWD resistance and spleen-index phenotypes from five year-classes of rainbow trout (Data S1). Complete dataset of spleen index values of fish used in QTL analyses (Data S2). Summary of BCWD phenotype data used in QTL analyses (Data S3). Combined genetic map for SI and BCWD QTL maps listing Omy Chr, markers, recombination and chromosome location in relative distance, cM (Data S4).
(XLSX)

## References

[1] Barnes ME, Brown ML (2011) A review of *Flavobacterium psychrophilum* biology, clinical signs, and bacterial cold water disease prevention and treatment. The Open Fish Sci J 4: 1–9.

[2] Nematollahi A, Decostere A, Pasmans F, Haesebrouck F (2003) *Flavobacterium psychrophilum* infections in salmonid fish. J Fish Dis 26: 563–574. 14653314 10.1046/j.1365-2761.2003.00488.x

[3] Starliper CE (2011) Bacterial coldwater disease of fishes caused by *Flavobacterium psychrophilum* . J Adv Res 2: 97–108.

[4] Groff JM, LaPatra SE (2000) Infectious diseases impacting the commercial culture of salmonids. J Appl Aquacult 10: 17–90.

[5] Brown LL, Cox WT, Levine RP (1997) Evidence that the causal agent of bacterial coldwater disease *Flavobacterium psychrophilum* is transmitted within salmonid eggs. Dis Aquat Org 29: 213–218.

[6] Cipriano RC (2005) Intraovum infection caused by *Flavobacterium psychrophilum* among eggs from captive Atlantic salmon broodfish. J Aquat Animal Health 17: 275–283.

[7] Vatsos IN, Thompson KD, Adams A (2006) Colonization of rainbow trout, *Oncorhynchus mykiss* (Walbaum), eggs by *Flavobacterium psychrophilum*, the causative agent of rainbow trout fry syndrome. J Fish Dis 29: 441–444. 16866929 10.1111/j.1365-2761.2006.00735.x

[8] Henryon M, Berg P, Olesen NJ, Kjaer TE, Slierendrecht WJ, et al (2005) Selective breeding provides an approach to increase resistance of rainbow trout (*Onchorhynchus mykiss*) to the diseases, enteric redmouth disease, rainbow trout fry syndrome, and viral haemorrhagic septicaemia. Aquaculture 250 n3–4: 621–636.

[9] Leeds TD, Silverstein JT, Weber GM, Vallejo RL, Palti Y, et al (2010) Response to selection for bacterial cold water disease resistance in rainbow trout. J Anim Sci 88: 1936–1946. 20154172 10.2527/jas.2009-2538

[10] Silverstein JT, Vallejo RL, Palti Y, Leeds TD, Rexroad CE 3rd, et al (2009) Rainbow trout resistance to bacterial cold-water disease is moderately heritable and is not adversely correlated with growth. J Anim Sci 87: 860–867. 19028851 10.2527/jas.2008-1157

[11] Hadidi S, Glenney GW, Welch TJ, Silverstein JT, Wiens GD (2008) Spleen size predicts resistance of rainbow trout to *Flavobacterium psychrophilum* challenge. J Immunol 180: 4156–4165. 18322227 10.4049/jimmunol.180.6.4156

[12] Wiens GD, LaPatra SE, Welch TJ, Evenhuis JP, Rexroad CE III, et al (2013) On-farm performance of rainbow trout (*Oncorhynchus mykiss*) selectively bred for resistance to bacterial cold water disease: Effect of rearing environment on survival phenotype. Aquaculture. 388–391: 128–136.

[13] Crump EM, Perry MB, Clouthier SC, Kay WW (2001) Antigenic characterization of the fish pathogen *Flavobacterium psychrophilum* . Appl and Environ Micro 67: 750–759. 10.1128/AEM.67.2.750-759.2001 PMC92644 11157240

[14] LaFrentz BR, LaPatra SE, Jones GR, Cain KD (2003) Passive immunization of rainbow trout, *Oncorhynchus mykiss* (Walbaum), against *Flavobacterium psychrophilum*, the causative agent of bacterial coldwater disease and rainbow trout fry syndrome. J Fish Dis 26: 371–384. 12946006 10.1046/j.1365-2761.2003.00468.x

[15] LaFrentz BR, LaPatra SE, Jones GR, Cain KD (2004) Protective immunity in rainbow trout *Oncorhynchus mykiss* following immunization with distinct molecular mass fractions isolated from *Flavobacterium psychrophilum* . Dis Aquat Organ 59: 17–26. 15212288 10.3354/dao059017

[16] Vallejo RL, Wiens GD, Rexroad CE 3rd, Welch TJ, Evenhuis JP, et al (2010) Evidence of major genes affecting resistance to bacterial cold water disease in rainbow trout using Bayesian methods of segregation analysis. J Anim Sci 88: 3814–3832. 20833766 10.2527/jas.2010-2951

[17] Boldman KG, Kriese LA, Van Vleck LD, Van Tassell CP, Kachman SD (1995) A manual for the use of MTDFREML. A set of programs to obtain estimates of variances and covariances [Draft]. Clay Center, NE: USDA-ARS.

[18] Falconer DS, Mackay TFC (1996) Introduction to quantitative genetics. 4^th^ Ed. HarlowUK: Pearson Education Limited. 464 p.

[19] Vallejo RL, Rexroad CE 3rd, Silverstein JT, Janss LL, Weber GM (2009) Evidence of major genes affecting stress response in rainbow trout using Bayesian methods of complex segregation analysis. J Anim Sci 87: 3490–3505. 19648504 10.2527/jas.2008-1616

[20] Janss LLG (2008) iBay manual version 1.46. Leiden, Netherlands: Janss Biostatistics, P.O. Box 535.

[21] Rexroad CE 3rd, Palti Y, Gahr SA, Vallejo RL (2008) A second generation genetic map for rainbow trout (*Oncorhynchus mykiss*). BMC Genet 9: 74. 19019240 10.1186/1471-2156-9-74 PMC2605456

[22] Palti Y, Genet C, Luo MC, Charlet A, Gao G, et al (2011) A first generation integrated map of the rainbow trout genome. BMC Genomics 12: 180. 21473775 10.1186/1471-2164-12-180 PMC3079668

[23] SAS (2007) SAS 9.1.3 Help and Documentation. Cary, NC: SAS Institute Inc.

[24] Seaton G, Hernandez J, Grunchec JA, White I, Allen J, et al. GRIDQTL: A grid portal for QTL mapping of computer intensive datasets; 2006 August 13–18, 2006; Belo Horizonte, MG, Brazil.

[25] Knott SA, Elsen JM, Haley CS (1996) Methods for multiple-marker mapping of quantitative trait loci in half-sib populations. Theoret Appl Genet 93: 71–80. 24162201 10.1007/BF00225729

[26] Lynch M, Walsh B (1997) Genetics and analysis of quantitative traits. Sunderland, MA: Sinauer Associates Inc.

[27] Leach RJ, Craigmile SC, Knott SA, Williams JL, Glass EJ (2010) Quantitative trait loci for variation in immune response to a Foot-and-Mouth Disease virus peptide. BMC Genet 11. 10.1186/1471-2156-11-107 PMC3019142 21138580

[28] Hu ZL, Park CA, Wu XL, Reecy JM (2013) Animal QTLdb: an improved database tool for livestock animal QTL/association data dissemination in the post-genome era. Nucleic Acids Res 41: D871–879. 23180796 10.1093/nar/gks1150 PMC3531174

[29] Palti Y, Genet C, Gao G, Hu Y, You FM, et al (2012) A second generation integrated map of the rainbow trout (*Oncorhynchus mykiss*) genome: analysis of conserved synteny with model fish genomes. Mar Biotechnol (NY) 14: 343–357. 22101344 10.1007/s10126-011-9418-z

[30] Gjedrem T (2010) The first family-based breeding program in aquaculture. Reviews in Aquaculture 2: 2–15.

[31] Kjøglum S, Henryon M, Aasmundstad T, Korsgaard I (2008) Selective breeding can increase resistance of Atlantic salmon to furunculosis, infectious salmon anaemia and infectious pancreatic necrosis. Aquacult Res 39: 498–505.

[32] Moen T (2010) Breeding for resistance to viral diseases in salmonids. In: Bishop SC, Axford, R.F.E, Nicholas, F.W, Owen, J.B., editor. Breeding for Disease Resistance in Farm Animals. 3rd ed. Oxforshire, UK: CABI. 166–179.

[33] Stear MJ, Nikbakht G, Matthews L, Jonsson NN (2012) Breeding for disease resistance in livestock and fish. CAB Reviews: Perspectives in Agriculture, Veterinary Science, Nutrition and Natural Resources 7. 10.1079/pavsnnr202116039 PMC8580373 34765015

[34] Brendolan A, Rosado MM, Carsetti R, Selleri L, Dear TN (2007) Development and function of the mammalian spleen. Bioessays 29: 166–177. 17226804 10.1002/bies.20528

[35] Fänge R, Nilsson S (1985) The fish spleen: structure and function. Experientia 41: 152–158. 3972063 10.1007/BF02002607

[36] Van Muiswinkel WB, Lamers CH, Rombout JH (1991) Structural and functional aspects of the spleen in bony fish. Res Immunol 142: 362–366. 1925007 10.1016/0923-2494(91)90093-x

[37] Visscher PM, Hill WG, Wray NR (2008) Heritability in the genomics era–concepts and misconceptions. Nat Rev Genet 9: 255–266. 18319743 10.1038/nrg2322

[38] Figenschou L, Folstad I, Rudolfsen G, Hanssen SA, Kortet R, et al.. (2012) The relative effect of parasites and social status on sperm traits in Arctic charr. Behav Ecol: 1–8.

[39] Wiens GD, Vallejo RL (2010) Temporal and pathogen-load dependent changes in rainbow trout (*Oncorhynchus mykiss*) immune response traits following challenge with biotype 2 Yersinia ruckeri. Fish Shellfish Immunol 29: 639–647. 20600959 10.1016/j.fsi.2010.06.010

[40] Fawcett GL, Roseman CC, Jarvis JP, Wang B, Wolf JB, et al (2008) Genetic architecture of adiposity and organ weight using combined generation QTL analysis. Obesity (Silver Spring) 16: 1861–1868. 18551125 10.1038/oby.2008.300

[41] Kenney-Hunt JP, Vaughn TT, Pletscher LS, Peripato A, Routman E, et al (2006) Quantitative trait loci for body size components in mice. Mamm Genome 17: 526–537. 16783635 10.1007/s00335-005-0160-6

[42] Nichols KM, Edo AF, Wheeler PA, Thorgaard GH (2008) The genetic basis of smoltification-related traits in *Oncorhynchus mykiss* . Genetics 179: 1559–1575. 18562654 10.1534/genetics.107.084251 PMC2475755

[43] Wringe BF, Devlin RH, Ferguson MM, Moghadam HK, Sakhrani D, et al (2010) Growth-related quantitative trait loci in domestic and wild rainbow trout (*Oncorhynchus mykiss*). BMC Genet 11: 63. 20609225 10.1186/1471-2156-11-63 PMC2914766

[44] Palti Y, Luo MC, Hu Y, Genet C, You FM, et al (2009) A first generation BAC-based physical map of the rainbow trout genome. BMC Genomics 10: 462. 19814815 10.1186/1471-2164-10-462 PMC2763887

[45] Palti Y, Rodriguez MF, Vallejo RL, Rexroad CE 3rd (2006) Mapping of Toll-like receptor genes in rainbow trout. Anim Genet 37: 597–598. 17121610 10.1111/j.1365-2052.2006.01527.x

[46] Phillips RB, Ventura AB, Dekoning JJ, Nichols KM (2012) Mapping rainbow trout immune genes involved in inflammation reveals conserved blocks of immune genes in teleosts. Anim Genet 44: 107–113. 23013476 10.1111/j.1365-2052.2011.02314.x

[47] Phillips RB, Zimmerman A, Noakes MA, Palti Y, Morasch MR, et al (2003) Physical and genetic mapping of the rainbow trout major histocompatibility regions: evidence for duplication of the class I region. Immunogenetics 55: 561–569. 14566436 10.1007/s00251-003-0615-4

[48] Wagner GP, Zhang J (2011) The pleiotropic structure of the genotype-phenotype map: the evolvability of complex organisms. Nat Rev Genet 12: 204–213. 21331091 10.1038/nrg2949

[49] Solovieff N, Cotsapas C, Lee PH, Purcell SM, Smoller JW (2013) Pleiotropy in complex traits: challenges and strategies. Nature Rev Genet 14: 483–495. 23752797 10.1038/nrg3461 PMC4104202

[50] Quillet E, Dorson M, Aubard G, Torhy C (2007) In vitro assay to select rainbow trout with variable resistance/susceptibility to viral haemorrhagic septicaemia virus. Dis Aquat Org 76: 7–16. 17718160 10.3354/dao076007

[51] Verrier ER, Langevin C, Tohry C, Houel A, Ducrocq V, et al (2012) Genetic resistance to rhabdovirus infection in teleost fish is paralleled to the derived cell resistance status. PLoS One 7: e33935. 22514610 10.1371/journal.pone.0033935 PMC3326022

[52] Kube PD, Taylor RS, Elliott NG (2012) Genetic variation in parasite resistance of Atlantic salmon to amoebic gill disease over multiple infections. Aquaculture 364–365: 165–172.

[53] Taylor RS, Kube PD, Muller WJ, Elliott NG (2009) Genetic variation of gross gill pathology and survival of Atlantic salmon (*Salmo salar* L.) during natural amoebic gill disease challenge. Aquaculture 294: 172–179.

[54] Langevin C, Blanco M, Martin SA, Jouneau L, Bernardet JF, et al (2012) Transcriptional responses of resistant and susceptible fish clones to the bacterial pathogen *Flavobacterium psychrophilum* . PLoS One 7: e39126. 22720048 10.1371/journal.pone.0039126 PMC3374740

[55] Baerwald MR, Petersen JL, Hedrick RP, Schisler GJ, May B (2010) A major effect quantitative trait locus for whirling disease resistance identified in rainbow trout (*Oncorhynchus mykiss*). Heredity 106: 920–926. 21048672 10.1038/hdy.2010.137 PMC3186244

[56] Houston RD, Gheyas A, Hamilton A, Guy DR, Tinch AE, et al (2008) Detection and confirmation of a major QTL affecting resistance to infectious pancreatic necrosis (IPN) in Atlantic salmon (*Salmo salar*). Dev Biol (Basel) 132: 199–204. 18817302 10.1159/000317160

[57] Houston RD, Haley CS, Hamilton A, Guy DR, Mota-Velasco JC, et al (2010) The susceptibility of Atlantic salmon fry to freshwater infectious pancreatic necrosis is largely explained by a major QTL. Heredity 105: 318–327. 19935825 10.1038/hdy.2009.171

[58] Moen T, Baranski M, Sonesson AK, Kjoglum S (2009) Confirmation and fine-mapping of a major QTL for resistance to infectious pancreatic necrosis in Atlantic salmon (*Salmo salar*): population-level associations between markers and trait. BMC Genom 10: 368. 10.1186/1471-2164-10-368 PMC2728743 19664221

[59] Moen T, Sonesson AK, Hayes B, Lien S, Munck H, et al (2007) Mapping of a quantitative trait locus for resistance against infectious salmon anaemia in Atlantic salmon (*Salmo salar*): comparing survival analysis with analysis on affected/resistant data. BMC Genet 8: 53. 17697344 10.1186/1471-2156-8-53 PMC2000910

[60] Verrier ER, Dorson M, Mauger S, Torhy C, Ciobotaru C, et al (2013) Resistance to a Rhabdovirus (VHSV) in Rainbow Trout: Identification of a major QTL related to innate mechanisms. PLoS One 8: e55302. 23390526 10.1371/journal.pone.0055302 PMC3563530

[61] Darvasi A, Soller M (1992) Selective genotyping for determination of linkage between a marker locus and a quantitative trait locus. Theoret Appl Genet 85: 353–359. 24197326 10.1007/BF00222881

