# Supplementary material for: Assessment of Genetic Correlation between Bacterial Cold Water Disease Resistance and Spleen Index in a Domesticated Population of Rainbow Trout: Identification of QTL on Chromosome Omy19
Source: PLoS One. 2013 Oct 9;8(10):e75749. doi: 10.1371/journal.pone.0075749 (PMC3794016; doi:10.1371/journal.pone.0075749)
Supplement: File S1 — Tables S1–S6. Rainbow trout year class, body weight, age and dose of challenge with F. psychrophilum strain CSF259-93 (Table S1). Measurement of spleen size in odd-year and even-year line rainbow trout populations (Table S2). Predicted major gene genotypes for parents of rainbow trout families after Bayesian segregation analysis1 of ln(spleen index) (Table S3). Basic statistical measures for spleen related traits in rainbow trout from cross 2008132 (Table S4). Genotyped and mapped microsatellite markers per rainbow trout chromosome (Table S5). Significant and suggestive QTL for body weight using half-sib family regression analysis in rainbow trout family 2008132 (Table S6). (DOCX) [file pone.0075749.s001.docx]

**Supplemental Information File_S1**

**(Tables S1-S6)**

Assessment of genetic correlation between bacterial cold water disease resistance and spleen index in a domesticated population of rainbow trout: Identification of QTL on chromosome Omy19.

Gregory D. Wiens, Roger L. Vallejo, Timothy D. Leeds, Yniv Palti, Sima Hadidi, Sixin Liu, Jason P. Evenhuis, Timothy J. Welch, and Caird E. Rexroad III

**Table S1.** Rainbow trout year class**,** body weight, age and dose of challenge with *F. psychrophilum* strain CSF 259-93.

|  |  | **Body weight (BW)** | | |  | | ***Fp* challenge** | | | | | | | | | |
| --- | --- | --- | --- | --- | --- | --- | --- | --- | --- | --- | --- | --- | --- | --- | --- | --- |
| **Population**  **(Year)** | | **Full-sib families,**  **n** | **Mean**  **Age^1^,**  **d** | **Mean**  **BW,**  **g (SD)** |  | | **Total**  **Fish,**  **n** | | **Mean**  **Age^1^,**  **d** | **Date**  **(challenge**  **#)** | **Inj.**  **Vol.,**  **μL** | **Challenge**  **Dose,**  **CFU fish^-1^** | **CFU g^-1^ Mean**  **BW** | **Tanks**  **(fish/**  **tank)** | **Tank size (L)** | **Water Temp.,**  **^o^C** |
| **Odd-year line^2^** | |  |  |  |  | |  | |  |  |  |  |  |  |  |  |
| G_0_ (2005) | | 71 | 68 | 2.4±0.3 |  | | 4,496 | | 82 | 5/24/05 (1) | 25 | 8.75 x 10^6^ | 3.8 x 10^6^ | 1-2(40) | 2.4L | 13.0±0.5 |
| G_1_ (2007) | | 97 | 72 | 2.4±0.3 |  | | 5,782 | | 85 | 5/14/07 (2) | 25 | 6.5 x 10^6^ | 2.7 x 10^6^ | 1-3(40) | 2.4L | 13.1±0.1 |
| F_1_ QTL (2007) | | 15 | 104 | 9.7±0.9 |  | | 2,960 | | 118 | 6/15/07 (3) | 100 | 2.7 x 10^7^ | 2.8 x 10^6^ | 4 (50) | 15L | 13.1±0.2 |
| G_2_  (2009) | | 114 | 84 | 4.1±0.9 |  | | 6,512 | | 84 | 6/2/09 (6) | 50 | 1.2 x 10^7^ | 2.9 x 10^6^ | 1-2(40) | 2.4L | 12.9±0.3 |
| F_2_ QTL#1 (2009) | | 10 | 82 | 3.0±0.3 |  | | 1,994 | | 82 | 4/30/09 (5) | 25 | 1.4 x 10^6^ | 0.5 x 10^6^ | 5 (40) | 2.4L | 12.6±0.1 |
| F_2_ QTL#2a (2009) | | 5 | 124 | 11.7±1.3 |  | | 973 | | 124 | 7/6/09 (7) | 100 | 4.9 x 10^6^ | 0.4 x 10^6^ | 4 (50) | 10L | 13.0±0.1 |
| F_2_ QTL#2b (2009) | | 3 | 149 | 21.7±3.1 |  | | 178 | | 149 | 7/6/09 (7) | 100 | 4.9 x 10^6^ | 0.2 x 10^6^ | 2 (30) | 10L | 13.0±0.1 |
| ARS-Fp-R  and ARS-Fp-S  (2009) | | 10 | 123 | 13.1±1.8 |  | | 692 | | 123 | 7/6/09 (7) | 100 | 4.9 x 10^6^ | 0.4 x 10^6^ | 2 (35) | 10L | 13.0±0.1 |
| **Even-year line^3^** | |  |  |  |  | |  | |  |  |  |  |  |  |  |  |
| G_0_ (2006) | | 12  (3 grp^4^) | 173 | 39.6± 11.6 |  | | 360 | | 190 | 8/15/05 | 100 | 2.1 x 10^7^ | 0.5 x 10^6^ | 3 (40) | 100L | 13.5±0.2 |
| F_1_ SI QTL (2008) | | 15 | 114 | 5.0±0.8 |  | | 1,782 | | 114 | 6/6/08 (4) | 25 | 8.2 x 10^6^ | 1.6 x 10^6^ | 3 (40) | 15L | 12.7±0.3 |
| **Total families^5^**  **and fish 2005-2009** | | 322 |  |  |  | | 25,369 | |  |  |  |  |  |  |  |  |

^1^Age is measured in days after hatching.

^2^Parents of the 2005 odd-year line were from the NCCCWA 2003 population and a limited number were 3 year-old from the NCCCWA 2002 population. Generation zero (G_0_) was the first generation of BCWD evaluation.

^3^Parents of the 2006 even-year line were from our 2004 growth selected population with pedigree information back to 2002. The 2006 year class was generation zero (G_0_) with respect to even-year spleen-size evaluation.

^4^Data were not used in heritability and genetic correlation analyses as four families were pooled into three groups prior to challenge.

^5^Represents number of unique families assayed. Fish from eighteen families were included in more than one challenge experiment.

**Table S2.** Measurement of spleen size in odd-year and even-year line rainbow trout populations.

| **Population**  **(year)** | **Full-sib Families, n** | **Total**  **Fish,**  **n** | **Mean Age at Spleen measure,**  **d** | **Mean**  **BW,**  **g (**±1 **SD)** |
| --- | --- | --- | --- | --- |
| **Odd-year line** |  |  |  |  |
| G_0_ (2005) | 8 | 120 | 96 | 10.2±2.5 |
| G_1_ Population and F_1_ BCWD QTL (2007) | 107 | 1944 | 128 | 22.2±5.6 |
| ARS-Fp-R and ARS-Fp-S (2009) | 10 | 200 | 150 | 31.5±6.8 |
| F_2_ BCWD QTL (2009) | 11 | 438 | 238 | 144±51 |
| **Even-year line** |  |  |  |  |
| G_0_ (2006) | 100 | 300 | 123 | 25.2±7.7 |
| G_0_ selected crosses^1^ (2006) | 12 | 340 | 174 | 40.0±11.5 |
| F_1_ SI QTL (2008) | 15 | 2303 | 145 to 633 | 6 to 3716 |
| **Total families and fish 2005-2009** | 251 | 5645 |  |  |

**^1^**Families were selected from the G_0_ (2006, population) and re-evaluated.

**Table S3.** Predicted major gene genotypes for parents of rainbow trout families after Bayesian segregation analysis^1^ of *ln*(spleen index).

| **Family**  **ID** | **Parent ID** | **Sex^2^** | **Spleen weight (mg)** | **Spleen index** | **$g_{i}$^3^** | **$PSD\left(g_{i}\right)$^4^** | **Accuracy^5^** | **Cross Type^6^** |
| --- | --- | --- | --- | --- | --- | --- | --- | --- |
| 2008090 | 14172 | 1 | 58.0 | 1.282 | 2 | 0.53 | 0.44 | Uninformative BC |
|  | 15343 | 2 | 48.1 | 1.503 | 1 | 0.35 | 0.75 |  |
| 2008098 | 15325 | 1 | 58.3 | 1.288 | 2 | 0.73 | -0.08 | Uninformative BC |
|  | 14945 | 2 | 46.7 | 1.458 | 1 | 0.34 | 0.77 |  |
| 2008109 | 14992 | 1 | 35.3 | 0.795 | 2 | 0.28 | 0.85 | F_2_ mapping family |
|  | 12795 | 2 | 24.3 | 0.615 | 2 | 0.09 | 0.99 |  |
| 2008132 | 13783 | 1 | 96.5 | 1.801 | 3 | 0.45 | 0.59 | BC mapping family |
|  | 14173 | 2 | 58.0 | 1.282 | 2 | 0.30 | 0.81 |  |
| 2008140 | 13889 | 1 | 35.8 | 0.673 | 2 | 0.41 | 0.66 | F_2_ mapping family |
|  | 12747 | 2 | 24.3 | 0.615 | 2 | 0.33 | 0.78 |  |
| 2008141 | 12750 | 1 | 24.3 | 0.615 | 2 | 0.38 | 0.72 | Uninformative BC |
|  | 15345 | 2 | 46.7 | 1.458 | 1 | 0.46 | 0.58 |  |
| 2008168 | 15347 | 1 | 46.7 | 1.458 | 2 | 0.04 | 1.00 | F_2_ mapping family |
|  | 14599 | 2 | 58.0 | 1.282 | 2 | 0.11 | 0.97 |  |
| 2008200 | 14707 | 1 | 58.0 | 1.282 | 2 | 0.39 | 0.69 | F_2_ mapping family |
|  | 13339 | 2 | 24.3 | 0.615 | 2 | 0.22 | 0.90 |  |
| 2008204 | 15094 | 1 | 35.3 | 0.795 | 2 | 0.48 | 0.53 | F_2_ mapping family |
|  | 14174 | 2 | 58.0 | 1.282 | 2 | 0.39 | 0.69 |  |
| 2008214 | 13690 | 1 | 96.5 | 1.801 | 2 | 0.49 | 0.51 | F_2_ mapping family |
|  | 12753 | 2 | 24.3 | 0.615 | 2 | 0.40 | 0.69 |  |
| 2008215 | 14943 | 1 | 46.7 | 1.458 | 2 | 0.42 | 0.64 | F_2_ mapping family |
|  | 12781 | 2 | 24.3 | 0.615 | 2 | 0.34 | 0.77 |  |
| 2008264 | 14944 | 1 | 46.7 | 1.458 | 1 | 0.38 | 0.72 | Uninformative BC |
|  | 14386 | 2 | 58.0 | 1.282 | 2 | 0.55 | 0.40 |  |
| 2008285 | 13296 | 1 | 21.6 | 0.735 | 3 | 0.31 | 0.81 | BC mapping family |
|  | 12800 | 2 | 24.3 | 0.615 | 2 | 0.00 | 1.00 |  |
| 2008286 | 15095 | 1 | 35.3 | 0.795 | 2 | 0.46 | 0.58 | F_2_ mapping family |
|  | 12890 | 2 | 21.6 | 0.735 | 2 | 0.52 | 0.47 |  |
| 2008299 | 12786 | 1 | 24.3 | 0.615 | 2 | 0.39 | 0.70 | F_2_ mapping family |
|  | 12809 | 2 | 21.6 | 0.735 | 2 | 0.75 | -0.12 |  |

^1^The best fitted mixed inheritance model (dominant spleen size-decreasing *A_1_* allele plus polygenic effects) from Bayesian segregation analysis was used to predict MG genotypes with iBay version 1.46 (Janss, 2008). The Gibbs sampler had these characteristics: number of iterations per chain = 1,200,000; burn-in period per chain = 600,000; thinning = 10,000; collected samples per chain = 60; total chains = 20; and total collected samples = 1,200.

^2^Sex of parents: 1 = male, and 2 = female.

^3^Posterior mean of a genotype for the *i^th^* animal, where $1=A_{1}A_{1}, 2=A_{1}A_{2},$ and $3=A_{2}A_{2}$.

^4^Posterior standard deviation of a genotype for the *i^th^* animal.

^5^Accuracy for the posterior mean of a genotype$\left(g_{i}\right)$.

^6^Cross type was defined using the predicted genotypes of parents within each family.

**Table S4.** Basic statistical measures for spleen related traits in rainbow trout from cross 2008132^1^.

| **Statistical measure** | **Body weight^2^** | **Spleen weight^2^** | **Spleen index^3^** | ***ln*(spleen index)^4^** |
| --- | --- | --- | --- | --- |
| N | 196 | 196 | 196 | 196 |
| Mean | 69.17 | 0.09 | 1.38 | 1.26 |
| Std. Deviation | 42.91 | 0.05 | 0.48 | 0.38 |
| Variance | 1840.89 | 0.00 | 0.24 | 0.14 |
| Std. Error | 3.06 | 0.00 | 0.03 | 0.03 |
| Coeff. Variation | 62.03 | 56.13 | 35.09 | 29.86 |
| Skewness | 2.63 | 1.52 | 0.25 | -0.40 |
| Kurtosis | 8.73 | 3.74 | -0.82 | -0.43 |
|  |  |  |  |  |
| **Normality test (*P*-value):** |  |  |  |  |
| Shapiro | <0.0001 | <0.0001 | <0.0001 | <0.0001 |
| Kolmogorov | <0.0100 | <0.0100 | <0.0100 | <0.0100 |
| Cramer | <0.0050 | <0.0050 | <0.0050 | <0.0050 |
| Anderson | <0.0050 | <0.0050 | <0.0050 | <0.0050 |

^1^A random sample of *N* = 300 offspring from full-sib family 2008132 was evaluated for spleen weight and body weight. For the QTL genome scan study, about 1/3 of fish from the upper and lower tail distribution for spleen index were selected for marker genotyping (*n* = 196, selective genotyping).

^2^Spleen weight and body weight per individual animal; measured in grams.

^3^Spleen index was defined as $SI= \left( {Spleen weight}/{Body weight} \right)*1000.$

^4^Spleen index was transformed using a natural logarithm of SI (log base e).

**Table S5.** Genotyped^1^ and mapped^2^ microsatellite markers per rainbow trout chromosome.

| **Chromosome** | | | |  | **Marker information content^4^** | | | | |
| --- | --- | --- | --- | --- | --- | --- | --- | --- | --- |
|  |  |  |  |  | **Dam families** | |  | **Sire families** | |
| **Number^3^** | **Number of markers** | **Length (cM)** | **Average marker density** |  | **Mean** | **SD** |  | **Mean** | **SD** |
| 1 | 8 | 45.0 | 5.6 |  | 0.87 | 0.10 |  | 0.95 | 0.04 |
| 2 | 11 | 89.1 | 8.1 |  | 0.85 | 0.08 |  | 0.67 | 0.29 |
| 3 | 14 | 75.5 | 5.4 |  | 0.90 | 0.07 |  | 0.85 | 0.17 |
| 4 | 10 | 88.8 | 8.9 |  | 0.83 | 0.09 |  | 0.66 | 0.26 |
| 5 | 11 | 101.8 | 9.3 |  | 0.81 | 0.15 |  | 0.71 | 0.17 |
| 6 | 11 | 66.2 | 6.0 |  | 0.88 | 0.07 |  | 0.92 | 0.08 |
| 7 | 17 | 101.0 | 5.9 |  | 0.91 | 0.08 |  | 0.87 | 0.10 |
| 8 | 13 | 78.8 | 6.1 |  | 0.90 | 0.08 |  | 0.91 | 0.12 |
| 9 | 14 | 89.3 | 6.4 |  | 0.88 | 0.11 |  | 0.88 | 0.13 |
| 10 | 18 | 99.6 | 5.5 |  | 0.83 | 0.15 |  | 0.83 | 0.17 |
| 11 | 11 | 40.6 | 3.7 |  | 0.89 | 0.07 |  | 0.98 | 0.01 |
| 12 | 14 | 80.8 | 5.8 |  | 0.81 | 0.15 |  | 0.87 | 0.14 |
| 13 | 8 | 61.7 | 7.7 |  | 0.70 | 0.24 |  | 0.79 | 0.15 |
| 14 | 10 | 64.3 | 6.4 |  | 0.90 | 0.05 |  | 0.89 | 0.12 |
| 15 | 9 | 89.0 | 9.9 |  | 0.83 | 0.09 |  | 0.65 | 0.27 |
| 16 | 8 | 44.4 | 5.6 |  | 0.82 | 0.09 |  | 0.80 | 0.14 |
| 17 | 15 | 112.8 | 7.5 |  | 0.85 | 0.13 |  | 0.83 | 0.12 |
| 18 | 9 | 70.8 | 7.9 |  | 0.80 | 0.13 |  | 0.73 | 0.25 |
| 19 | 14 | 63.2 | 4.5 |  | 0.90 | 0.06 |  | 0.82 | 0.16 |
| 20 | 8 | 40.1 | 5.0 |  | 0.92 | 0.05 |  | 0.93 | 0.08 |
| 21 | 12 | 60.3 | 5.0 |  | 0.91 | 0.06 |  | 0.85 | 0.14 |
| 22 | 10 | 59.9 | 6.0 |  | 0.81 | 0.13 |  | 0.82 | 0.14 |
| 23 | 6 | 44.5 | 7.4 |  | 0.86 | 0.07 |  | 0.81 | 0.17 |
| 24 | 6 | 36.3 | 6.1 |  | 0.91 | 0.04 |  | 0.89 | 0.11 |
| 25 | 10 | 103.6 | 10.4 |  | 0.79 | 0.15 |  | 0.77 | 0.12 |
| 26 | 10 | 26.7 | 2.7 |  | 0.65 | 0.29 |  | 0.73 | 0.20 |
| 27 | 7 | 26.5 | 3.8 |  | 0.84 | 0.11 |  | 0.98 | 0.01 |
| 28 | 6 | 75.8 | 12.6 |  | 0.61 | 0.26 |  | 0.78 | 0.14 |
| sex | 7 | 67.4 | 9.6 |  | 0.70 | 0.22 |  | 0.84 | 0.12 |
| Total/Average | 307 | 2003.8 | 6.5 |  | 0.83 | 0.12 |  | 0.83 | 0.14 |

^1^A panel of 341 microsatellite markers were genotyped using the QTL mapping family.

^2^Genetic maps were developed with software MULTIMAP version 2.0 (Matise et al., 1994).

^3^Chromosome number is based on Rexroad et al. (2008).

^4^ Marker information content combines the multilocus probability of individuals inheriting allele 1 or 2 from the common parent with marker segregation distortion (Knott et al., 1996).

**Table S6.** Significant and suggestive QTL for body weight using half-sib family regression analysis^1^ in rainbow trout family 2008132.

| **Omy^2^** | **cM^3^** | **LR^4^** | ***F*-value^5^** | ***F_ChromWide_ _P=0.05_* ^6^** | ***P_ExperWide P=0.05_* ^7^** | $\boldsymbol{h}_{\boldsymbol{q}}^{\boldsymbol{2}}$**^8^** | **95% C.I. ^9^** |  | **Closest marker^10^** | |
| --- | --- | --- | --- | --- | --- | --- | --- | --- | --- | --- |
|  |  |  |  |  |  |  |  |  | **Left** | **Right** |
| ***Dam HS family*** | | | | | | | | | | |
| 10 | 29 | 6.95 | 7.10* | 6.86 | 13.01 | 0.05 | 10-71 |  | OMM1332 | OMM1107 |
| ***Sire HS family*** | | | | | | | | | | |
| 10 | 0 | 12.40 | 12.84** | 6.09 | 11.94 | 0.09 | 0-19 |  | OMM1257 | OMM5267 |
| 22 | 26 | 8.08 | 8.27* | 5.83 | 11.94 | 0.05 | 0-45 |  | OMM1625 | OMM1365 |
| 29 | 67 | 6.27 | 6.39* | 5.53 | 11.94 | 0.04 | 0-67 |  | OMYRGT28TUF | OMM1026 |

^1^Footnotes are the same as Table 3.
